# N, N-Dimethyltryptamine (DMT), an Endogenous Hallucinogen: Past, Present, and Future Research to Determine Its Role and Function

**DOI:** 10.3389/fnins.2018.00536

**Published:** 2018-08-06

**Authors:** Steven A. Barker

**Affiliations:** Department of Comparative Biomedical Sciences, School of Veterinary Medicine, Louisiana State University, Baton Rouge, LA, United States

**Keywords:** N, N-dimethyltryptamine, hallucinogen, psychedelic, neurotransmitter, brain

## Abstract

This report provides a historical overview of research concerning the endogenous hallucinogen N, N-dimethyltryptamine (DMT), focusing on data regarding its biosynthesis and metabolism in the brain and peripheral tissues, methods and results for DMT detection in body fluids and brain, new sites of action for DMT, and new data regarding its possible physiological and therapeutic roles. Research that further elaborates its consideration as a putative neurotransmitter is also addressed. Taking these studies together, the report proposes several new directions and experiments to ascertain the role of DMT in the brain, including brain mapping of enzymes responsible for the biosynthesis of DMT, further studies to elaborate its presence and role in the pineal gland, a reconsideration of binding site data, and new administration and imaging studies. The need to resolve the “natural” role of an endogenous hallucinogen from the effects observed from peripheral administration are also emphasized.

## Introduction

Despite their presence in the human pharmacopeia for millennia, we have yet to resolve the biochemical mechanisms by which the hallucinogens (psychedelics) so dramatically alter perception and consciousness. It is the only class of compounds that efficiently and specifically does so. For that matter, we do not fully understand the biochemistry of perception itself or how we live such a vivid and complex internal life in the absence of external stimulation. We do not understand the basic biochemical mechanisms of some of our most common experiences, such as the many human aspects of creativity, imagination or dream states. This is also true for extraordinary states of consciousness such as “visions” or spontaneous hallucinations or phenomena such as near-death experiences (NDE). And it is troubling that we have not sufficiently turned the scientific method on these latter subjects despite the profound role they have played in the evolution of our science, philosophy, psychology and culture.

The experiences derived from the administration of hallucinogens are often compared to dream states. However, the experience of administered hallucinogenic substances is far more intense, robust and overwhelming than the subtlety of mere dreams. By comparison, the natural biochemical processes for our related “hallucinatory” experiences are obviously far more highly regulated, occurring as an orchestrated and inherent function of the “normal” brain. Nonetheless, it is conceivable that attaining an explanation for these related natural human phenomena may lie in resolving the biochemical mechanisms involved in the more dramatic pharmacology of hallucinogens, recognizing that the complexities and intensity of the “administered” experience are, essentially, an overdose relative to corresponding natural regulatory controls. Given their status as “psychedelics” (mind-manifesting substances), increased study of the hallucinogens, particularly with advanced brain imaging and molecular biology approaches, may provide a better understanding of the “common” biochemistry that creates mind.

**Figure 1:**
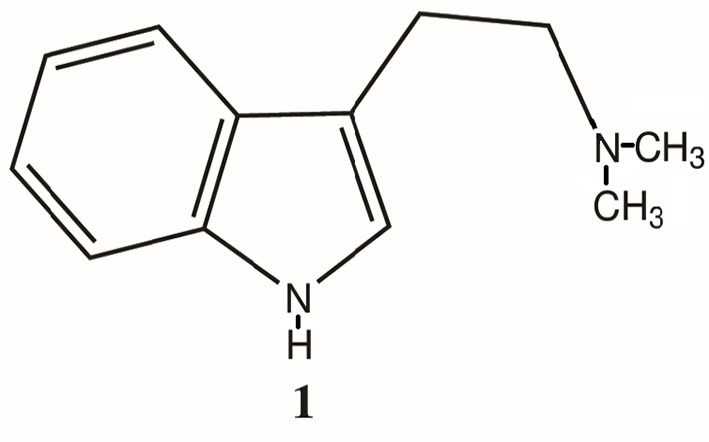
Structure of N, N-dimethyltryptamine (DMT, **1**).

Perhaps the science behind the discovery of endogenous opioids offers us a corollary. We came to better understand the common human experience of pain through examining the pharmacology of administered opiates and the subsequent discovery of endogenous opioid ligands, receptors and pathways that are predominantly responsible for and regulate the experience and perception of pain. Such may also be the case for understanding perception and consciousness. With the discovery of the endogenous hallucinogen N, N-dimethyltryptamine (DMT, **1**, Figure 1), perhaps, as with the endogenous opioids, we have a similar opportunity to understand perception and consciousness. Recent research has stimulated a renewed interest in further study of this compound as a neuro-regulatory substance and, thus, a potential neuro-pharmacological target. Taking results from these and more classical studies of DMT biochemistry and pharmacology together, this report examines some of the past and current data in the field and proposes several new directions and experiments to ascertain the role of endogenous DMT.

## A brief history of DMT

In terms of Western culture, DMT was first synthesized by a Canadian chemist, Richard Manske, in 1931 (Manske, 1931) but was, at the time, not assessed for human pharmacological effects. In 1946 the microbiologist Oswaldo Gonçalves de Lima discovered DMT's natural occurrence in plants (Goncalves de Lima, 1946). DMT's hallucinogenic properties were not discovered until 1956 when Stephen Szara, a pioneering Hungarian chemist and psychiatrist, extracted DMT from the Mimosa hostilis plant and administered the extract to himself intramuscularly (Szára, 1956). This sequence of events formed the link between modern science and the historical use of many DMT-containing plants as a cultural and religious ritual sacrament (McKenna et al., 1998), their effect on the psyche and the chemical structure of N, N-dimethyltryptamine.

The discovery of a number of hallucinogens in the 1950's and observations of their effects on perception, affect and behavior prompted hypotheses that the syndrome known as schizophrenia might be caused by an error in metabolism that produced such hallucinogens in the human brain, forming a schizo- or psycho-toxin (Osmond and Smythies, 1952). The presence of endogenous hallucinogenic compounds, related mainly to those resembling dopamine (mescaline) or serotonin (DMT), were subsequently sought. Although several interesting new compounds were found, the only known hallucinogens isolated were those derived from tryptophan (DMT, and 5-methoxy-DMT). Data were subsequently developed illustrating pathways for their endogenous synthesis in mammalian species, including humans. Over 60 studies were eventually undertaken in an attempt to correlate the presence or concentration of these compounds in blood and/or urine with a particular psychiatric diagnosis (for a review see Barker et al., 2012). However, there has yet to be any clear-cut or repeatable correlation of the presence or level of DMT in peripheral body fluids with any psychiatric diagnosis. Nonetheless, the discovery of endogenous hallucinogens and the possibilities rendered in various hypotheses surrounding their role and function in mental illness, normal and “extraordinary” brain function spurred further research into the mechanisms for their biosynthesis, metabolism and mode of action as well as for their known and profound effects on consciousness (Mishor et al., 2011; Araújo et al., 2015).

## DMT biosynthesis

**Figure 2:**
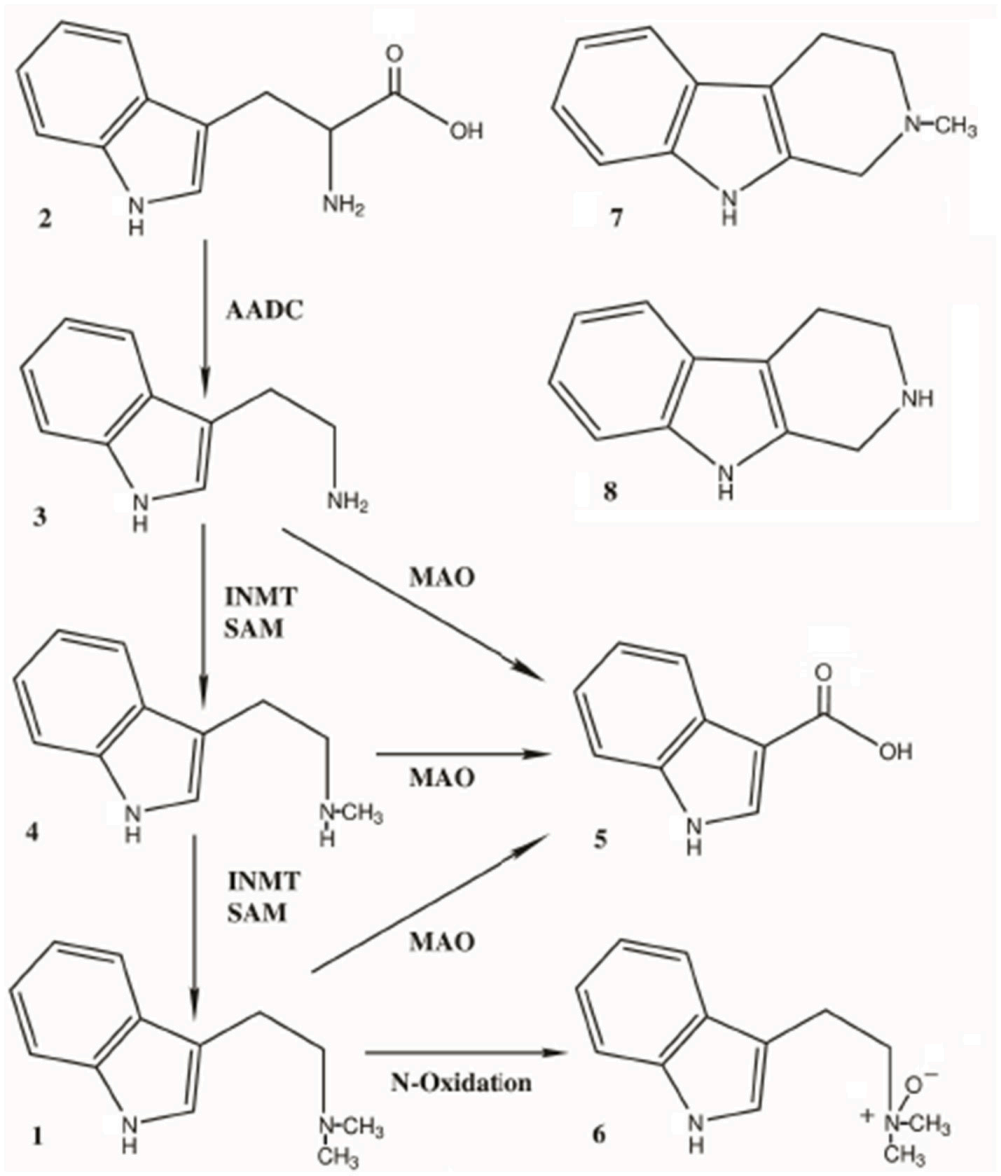
Pathways for the biosynthesis and metabolism of DMT, **1**. Biosynthesis: Tryptophan (**2**) is converted to tryptamine (TA, **3**) by aromatic amino acid decarboxylase (AADC). TA is dimethylated to first yield N-methyltryptamine (NMT, **4**) and then DMT (**1**) by indole-N-methyltransferase (INMT), using S-adenosyl-methionine (SAM) as the methyl source. Metabolism: TA, NMT and DMT are all substrates for monoamine oxidase, yielding indole-3-acetic acid (**5**, IAA) as both a common precursor metabolite and the most abundant metabolite of DMT itself. DMT is also converted to DMT-N-oxide (**6**) as the second-most abundant metabolite. Two 1,2,3,4-tetrahydro-beta-carbolines (THBCs) have also been identified as metabolites; 2-methyl-THBC (**7**, MTHBC) and THBC (**8**).

After the discovery of an indole-N-methyl transferase (INMT; Axelrod, 1961) in rat brain, researchers were soon examining whether the conversion of tryptophan (**2**, Figure 2) to tryptamine (TA; **3**, Figure 2) could be converted to DMT in the brain and other tissues from several mammalian species. Numerous studies subsequently demonstrated the biosynthesis of DMT in mammalian tissue preparations *in vitro* and *in vivo* (Saavedra and Axelrod, 1972; Saavedra et al., 1973). In 1972, Juan Saavedra and Julius Axelrod reported that intracisternally administered TA was converted to N-methyltryptamine (NMT; **4**, Figure 2) and DMT in the rat, the first demonstration of DMT's formation by brain tissue *in vivo*. Using dialyzed, centrifuged whole-brain homogenate supernatant from rats and humans, these same researchers determined that the rate of synthesis of DMT from TA was 350 and 450 pmol/g/hr and 250 and 360 pmol/g/h, using NMT as substrate, in these tissues, respectively. In 1973, Saavedra et al. characterized a nonspecific N-methyltransferase in rat and human brain, reporting a Km for the enzyme of 28 uM for TA as the substrate in rat brain. The highest enzyme activity in human brain was found in the subcortical layers of the fronto-parietal and temporal lobes and the cortical layers of the frontal parietal lobe. However, an INMT found in rabbit lung was shown to have a much higher Km (270 uM, Thompson and Weinshilboum, 1998; 340 uM, Raisanen and Karkkainen, 1978) than the brain enzyme in rats. This suggested that INMT may exist in several isoenzyme forms between species and possibly even within the same animal, each having different Km's and substrate affinities. INMT activity has subsequently been described in a variety of tissues and species. There have also been several reports of an endogenous inhibitor of INMT *in vivo* that may help regulate its activity and, thus, DMT biosynthesis (Wyatt et al., 1973a,b; Lin et al., 1974; Narasimhachari et al., 1974; Barker et al., 1981).

The combined data demonstrate that DMT is formed from tryptophan (**2**, Figure 2), a common dietary amino acid, via the enzyme aromatic L-amino acid decarboxylase (AADC) formation of TA (**3**, Figure 2) and its subsequent N, N-dimethylation. The enzyme indolethylamine-N-methyltransferase (INMT) uses S-adenosyl-l-methionine as the methyl source to produce N-methyltryptamine (NMT; **4**, Figure 2) and then DMT (**1**, Figure 2). Both AADC and INMT act on other substrates as well. As a historical and research note regarding DMT, there was initial confusion and misidentification of the products formed when using 5-methyltetrahydrofolate (5-MTHF) as the methyl source in INMT studies due to formation of indole-ethylamine condensation products with formaldehyde (tetrahydro-beta-carbolines) (Barchas et al., 1974; Lin and Narasimhachari, 1974; Rosengarten and Friedhoff, 1976; Barker et al., 1981).

There has also been interest in the role of INMT and DMT biosynthesis in maturation and development. Relatively elevated levels of INMT activity have been found in the placenta from a variety of species, including humans (Thompson et al., 1999). INMT activity in rabbit lung was reported to be elevated in the fetus and to increase rapidly after birth, peaking at 15 days of age. It then declined to mature levels and remained constant through life (Lin et al., 1974). In this regard, Beaton and Morris (1984) have examined the ontogeny of DMT biosynthesis in the brain of neonatal rats and rats of various ages. Using gas chromatography-mass spectrometry with isotope dilution for their analyses, DMT was detected in the brain of neonatal rats from birth. DMT levels remained low (1–4 ng/g of whole brain tissue) until days 12 and 17 at which time they increased significantly and then returned to the initial low levels for all subsequent ages. There has yet to be any follow-on research as to the significance of this change in DMT concentrations during rat brain neurodevelopment or correlation with possible changes of INMT activity in other developing tissues, specifically during days 12–17. Nonetheless, these findings correlate well with the Lin et al. (1974) data for INMT changes in rabbits and deserve further inquiry.

There is a significant literature concerning INMT, particularly in peripheral tissues. INMT and its gene have been sequenced (Thompson et al., 1999), commercial antibodies for its detection have been developed and commercial probes exist for monitoring its mRNA and gene expression. A study using Northern blot detection of the INMT mRNA conducted by Thompson et al. (1999) in the rabbit suggested that INMT was present in significant quantities in the periphery, and particularly the lung, but that it was almost non-existent (low to absent) in the brain. These data became the foundation for several hypotheses that any neuropharmacological effects of endogenous DMT must lie in its formation in the periphery and its subsequent transport into the brain. This idea was strengthened by the fact that DMT has been shown to be readily, and perhaps actively, transported into the brain (Cozzi et al., 2009). However, the data concerning the apparent absence of INMT in brain would appear to be in conflict with the many earlier studies that demonstrated both *in vivo* and *in vitro* biosynthesis of DMT in the brain. Indeed, several studies had identified INMT activity or the enzyme itself in the central nervous system (CNS) including the medulla, the amygdala, uncus, and frontal cortex (Morgan and Mandell, 1969), the fronto-parietal and temporal lobes (Saavedra et al., 1973) and, more recently, the anterior horn of the spinal cord as well as the pineal gland (Cozzi et al., 2011).

Thus, in 2011, Cozzi et al. sought to determine why earlier studies (Thompson et al., 1999) had not detected significant INMT in brain using Northern blots despite several reports that brain tissue had been shown to synthesize DMT from TA. One possibility was that INMT was “expressed in nervous tissue but that in some situations, INMT mRNA is not detectable by Northern analysis (e.g., the INMT gene is inducible, INMT expression is limited to specific brain nuclei, or INMT mRNA in brain is short-lived).” Examining primate nervous system tissues (Rhesus macaque spinal cord, pineal gland, and retina) probed with rabbit polyclonal antibodies to human INMT, all three tissues tested positive. INMT immunoreactivity in spinal cord was found to be localized in ventral horn motoneurons. The study also showed that INMT response was “robust and punctuate” in the pineal gland. Further, intense INMT immunoreactivity was detected in retinal ganglion neurons and at synapses in the inner and outer plexiform layers (Cozzi et al., 2011). In 2012, Mavlyutov et al. reported that INMT is also localized in postsynaptic sites of C-terminals of rat motoneurons in close proximity to sigma-1 receptors, which have been linked to control of the activities of ion channels and G-protein-coupled receptors. It was proposed that the close association of INMT and sigma-1 receptors suggests that DMT is synthesized locally to effectively activate sigma-1 in motoneurons. It has been further proposed that DMT is an endogenous sigma-1 receptor regulator (Fontanilla et al., 2009; Su et al., 2009).

Taking these newer data together with historical *in vitro* and *in vivo* results regarding INMT enzyme activity in the brain and CNS, it is now clear that the work of Thompson and Weinshilboum (1998) is not the final word on DMT biosynthesis in the brain.

### Future research on the biosynthesis of DMT

Considering that tryptamine formation, itself a trace biogenic amine, is essential for the formation of DMT and given its own rapid metabolism by monoamine oxidase (MAO) as well, demonstrating its availability for the biosynthesis of DMT is also relevant to a complete elucidation of the overall pathway. Indeed, demonstrating the co-localization of AADC and INMT should be a necessary endeavor in any future research regarding DMT biosynthesis in both the brain and periphery. The colocalization of AADC in discreet brain cells and areas with INMT permits TA and, subsequently, DMT formation locally. With demonstration of colocalization of the necessary biosynthetic machinery in the brain, both AADC and INMT, mechanisms for a rapid biochemical response to signaling and DMT formation may be shown to exist. Furthermore, the demonstration of mechanisms for the protection, storage, release and reuptake of DMT would demonstrate that higher concentrations of DMT could be reached in the synaptic cleft and at neuronal receptors than would have to occur from, based on previous thought, formation and transport from the periphery. Pursuit of research of these mechanisms, as well as detailed mapping of INMT-AADC in the brain, is needed. We should not rule out the possibility that the biosynthesis and transport of DMT can and does occur from the periphery, however. Peripheral DMT, especially if synthesized in tissues that bypass liver metabolism on first pass, may also serve as a signaling compound from the periphery to the brain. Such signaling may occur in maintaining homeostasis or in response to extreme changes in physiology. However, the immediate availability of TA for the biosynthesis of DMT in the periphery should also be demonstrated and studies examining the co-localization of AADC and INMT in the periphery should also be performed. This will require using highly sensitive and well validated antibodies and probes for detection of INMT and/or its mRNA in brain and/or peripheral tissues as well as those for aromatic-L-amino acid decarboxylase (AADC). Demonstration of colocalization with AADC has not been previously conducted in any other study seeking to identify INMT's presence or to demonstrate INMT activity. Such a determination may prove fruitful since a preliminary examination for the possible colocalization of INMT and AADC in the brain is supported by the data provided in the Allen Brain Atlas, mapping INMT and AADC gene expression (brain-map.org).

A thorough re-examination of possible peripheral DMT biosynthesis is needed. Indeed, INMT actually methylates other substrates, such as histamine (Herman et al., 1985). Thus, much of the INMT in the periphery may be involved to a greater degree with methylation of other substances than TA alone. In this regard, *in vitro* studies of INMT as it relates to DMT biosynthesis necessarily added TA to their incubations, making TA “artificially” available in regions where natural levels may be absent or at significantly lower levels. Without a source for TA, the hypotheses regarding the formation of DMT in the periphery and its transport to the brain as a mechanism of action/function of endogenous DMT may be seen to be based on a less significant pathway than previously thought. Failure to demonstrate colocalization of INMT and AADC in the periphery would alter, to some degree, the focus of studies of peripheral synthesis and detection for understanding the role of endogenous DMT.

At least one study has now shown that the pineal gland has high concentrations of INMT (Cozzi et al., 2011). These data are underscored by the findings of Barker et al. (2013) demonstrating the presence of DMT in pineal perfusates from free-moving rats. Clearly, further research into the biosynthesis and role of DMT in the pineal is needed, as is a further assessment of our current knowledge of pineal function.

We will also need to examine protein and gene arrays to determine the factors that assist or work in concert with the up and down regulation of the INMT system in brain and how it responds to selected physiological changes. Such analyses will be essential in examining the possible role of DMT biosynthesis in changing biochemical and physiological events. We will also need to create brain-specific INMT KO animals, to further understand DMT biosynthesis and the “normal” role of DMT *in vivo*. It would also be of interest to better understand the possible role of DMT in neurodevelopment as suggested by the work of Beaton and Morris (1984) and Lin et al. (1974) in rats and rabbits, respectively. While DMT appears to clearly be biosynthesized in the pineal, mechanisms for its biosynthesis and release may exist in other brain areas as well and research into these other possibilities will also need to proceed.

## DMT metabolism

The metabolism of DMT has been thoroughly studied, with a great deal of newer data being provided from studies of ayahuasca administration (McIlhenny et al., 2012; Riba et al., 2012). All of the *in vivo* metabolism studies have shown that exogenously administered (IV, IM, smoking, etc.) DMT is rapidly metabolized and cleared, with only a small fraction of IV or IM administered DMT subsequently being found in urine. For example, 0.16% of an intramuscular dose of DMT was recovered as the parent compound following a 24 h urine collection (Kaplan et al., 1974). DMT administered in this manner reached a peak concentration in blood within 10–15 min and was below the limits of detection within 1 h. It was estimated that only 1.8% of an injected dose was present in blood at any one time. Due to rapid metabolism in the periphery, DMT is not orally active, being converted to inactive metabolites before sufficient penetration to the brain can occur (low bioavailability). DMT is only orally active if co-administered with a monoamine oxidase inhibitor (MAOI). DMT is pharmacologically active following administration by injection (intravenous or intramuscular routes) or smoking (vaporization and inhalation), pathways which can avoid first-pass metabolism by the liver to some degree (Riba et al., 2015). The time to onset of effects is rapid (seconds to minutes) by these routes and short lived (15–60 min depending on dose and route).

The primary route of metabolism for DMT (**1**, Figure 2) is via monoamine oxidase A (MAO-A), yielding indoleacetic acid (IAA; auxin; **5**, Figure 2). The other metabolites formed include DMT-N-oxide (DMT-NO; **6**, Figure 2), the second most abundant metabolite, and lesser amounts of N-methyltryptamine (NMT; **4**, Figure 2), which, along with TA, is also a substrate for MAO-A (Fish et al., 1955; Szara and Axelrod, 1959; Barker et al., 1980, 1981; Figure 2), with both yielding IAA. Inhibition of MAO leads to a shift in favor of the amounts of DMT-NO and NMT formed (Riba et al., 2003). Other metabolites have been reported, such as 6-hydroxy-DMT (6-OH-DMT), (Szára, 1961) as well as products from a peroxidase pathway, reported to yield N, N-dimethyl-N-formyl-kynuramine, and N, N-dimethyl-kynuramine (Tourino et al., 2013; Gomes et al., 2014). However, these latter metabolites have yet to be identified *in vivo*. Metabolites also result from the cyclization of an intermediate iminium ion that forms during demethylation of DMT, yielding 2-methyl- 1,2,3,4- tetrahydro-beta-carboline (MTHBC; **7**, Figure 2) and THBC (**8**, Figure 2; Barker et al., 1980, 1981).

**Figure 3:**
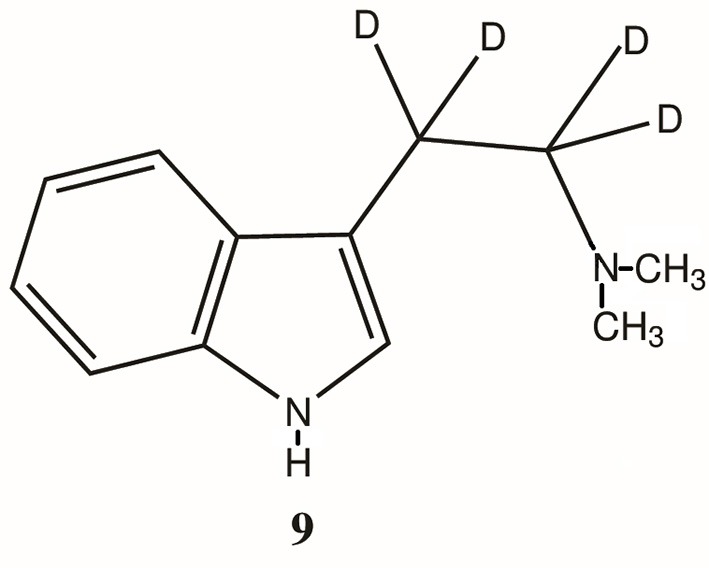
Structure of α, α, β, β-teradeutero-DMT (**9**). D = deuterium.

The primary role of MAO-A in the metabolism of DMT has been further confirmed by pretreatment of experimental subjects with the MAO inhibitor (MAOI) iproniazid as well as other MAOIs (Lu and Domino, 1974; Moore et al., 1975; Shah and Hedden, 1978; Barker et al., 1980, 1981), the ability of the MAO-inhibiting harmala alkaloids of ayahuasca to make DMT orally active (McKenna, 2004) and the increased half-life and extended effects of an α, α, β, β-tetradeutero-DMT (D_4_DMT; **9**, Figure 3), which is less susceptible to MAO-A metabolism due to the kinetic isotope effect (Barker et al., 1982, 1984; Beaton et al., 1982).

### Future research on the metabolism of DMT

While the metabolism of DMT has been thoroughly studied and a number of metabolites, both major and minor, have been identified (Figure 2), one of the complications in understanding the role and function of endogenous DMT has been the fact that, to date, no study examining body fluids (blood, urine, saliva) has ever been conducted to correlate such data with human physiological events, such as circadian changes, sex differences, etc. (Sitaram and McLeod, 1990; Barker et al., 2012). Of greater impact is the fact that, despite DMT's rapid metabolism and multiple metabolites, no study has fully assessed all of these compounds simultaneously to better understand DMT's overall occurrence or rate of endogenous synthesis, release, clearance and/or the overall assessment of the relevance of endogenous levels in the brain or periphery. All of these factors need to be examined. Given that peripherally administered DMT, at what must be considered as much higher doses than would be expected to occur naturally in the entire organism, is rapidly metabolized and cleared, measuring endogenous DMT alone in an attempt to assess its role and function is probably doomed to failure. This is particularly true if endogenous DMT is mainly produced, stored and metabolized in discreet brain areas and that DMT and its metabolites so produced never attain measurable levels in peripheral fluids. To the degree that DMT is produced peripherally, measurement of IAA, DMT-NO, N-methyltryptamine and the precursor for the synthesis of both DMT and NMT, tryptamine, would be advantageous. These compounds have been variously reported in tissue, blood and urine samples. However, this approach is complicated by the fact that the major MAO metabolite of all three of these latter compounds, IAA (Figure 2), is also derived from dietary sources and is produced from the action of bacteria in the gut. It is not unreasonable to question whether measurement of DMT and its metabolites, and thus the role and function of endogenous DMT, can be understood by simply trying to measure these compounds in the periphery. This is particularly true in understanding DMT production in the CNS. Peripheral measurements may not be the way to determine the central role of DMT and DMT produced in the brain may never be available for measurement in the periphery. Nonetheless, additional studies should determine if there is validity in such measurements and examine possible circadian, ultradian or diurnal variations in DMT synthesis as well as the changes that may occur due to alterations in other physiological parameters.

## DMT detection in blood, urine, and cerebrospinal fluid

Barker et al. (2012) have published a thorough overview of the 69 published studies examining blood, urine and cerebrospinal fluid detection of endogenous N, N-dimethylated tryptamines [N, N-dimethyltryptamine (DMT), 5-hydroxy-DMT (bufotenine, HDMT), and 5-methoxy-DMT (MDMT)]. Nearly all of the studies were directed at establishing a relationship between the presence and/or level of these compounds and a psychiatric diagnosis. In total, the 69 studies examined DMT in thousands of subjects. A critical review of these data determined, however, that most early studies reporting rather high concentrations of these compounds in blood and/or urine were most likely in error and any correlations based on these data were likewise probably incorrect. The reasons for this conclusion were: (1) Based on current analytical requirements for unequivocal structure identification, it is highly probable that many of these studies misidentified the target analyte. (2) If properly identified, the studies showed that a psychiatric diagnosis was not a necessary or sufficient criterion for finding one or more of these hallucinogens in various body fluids; “normal” controls were also positive (and sometimes higher) for these compounds. Nevertheless, it was also concluded that, particularly where mass spectral evidence was provided, DMT and HDMT are endogenous and can often be successfully measured in human body fluids. The evidence was less compelling for MDMT where the only two MS-based positive studies—in CSF—were performed by the same research group. There was no mass spectral data demonstrating detection of MDMT in blood or urine. There was also no study that attempted a determination of HDMT in CSF.

In conducting studies to determine the natural occurrence of a compound as being endogenous, it is also necessary to eliminate other possible dietary or environmental sources. Of the 69 studies reviewed, many addressed the possible source of DMT as being from diet or gut bacteria (Barker et al., 2012) by using special diets. Of those conducted, it was determined that neither was a source but additional research in this area using more modern technology and a more standard diet across studies is a necessity. There have also been only a few efforts to examine the many variables that may influence the levels of these compounds, such as circadian or diurnal variations, sleep stages and gender-age-related differences. Indeed, most of the studies collected only a single time point or were from 24 h collections (urine). Such infrequent sampling makes it impossible to assess central DMT production from peripheral measurements and suggests, perhaps incorrectly, that DMT only appears intermittently or not at all. In trying to compare the results, interpretations and correlation of the data were hampered by variability in sampling methods, amount of sample assayed, type of sample (plasma, serum and/or whole blood), divergent techniques and analytical methodology that also had highly variable or unspecified limits of detection.

### Future research measuring DMT in the blood, urine, and/or cerebrospinal fluid

In terms of pursuing future research on the presence of the endogenous indolealkylethylamines, further studies are necessary to determine whether MDMT actually exists in humans. Similarly, there are no data on the possible presence of HDMT in CSF although it has been routinely identified in urine (Barker et al., 2012). Future analyses to determine endogenous N, N-dimethyl-indolethylamines should also include a search for their major metabolites. The methodology applied in such analyses must include rigorous validated protocols for sample collection, storage, extraction and analyte stability and appropriate criteria for unequivocal detection and confirmation of the analytes using validated methods. Modern exact-mass liquid chromatography-mass spectrometry instrumentation should be the analytical method of choice. Such capabilities may then be applied to address the many variables that may influence the ability to measure DMT and/or its precursors and metabolites (Figure 2) in the periphery.

### Measurement of DMT in the brain

Many studies have been conducted to detect and/or quantitate DMT in blood and urine and only a few in the CSF of humans (Barker et al., 2012). However, the CSF studies made no effort to quantitate the DMT detected (Christian et al., 1975; Corbett et al., 1978; Smythies et al., 1979). In fact, there have been no efforts to quantify the actual levels of endogenous DMT and its metabolites in human brain and only a few have attempted to address the issue in rats. Barker et al. (2013) described the presence of DMT in pineal gland perfusates from free-moving rats but no quantitation was conducted since the perfusates were essentially dilutions of the surrounding tissue effluent and were collected at a single point-in-time. As noted, no circadian studies of DMT production or release from the pineal as a function of time have ever been conducted. In Kärkkäinen et al. (2005), using multiple extraction and clean-up steps and an LC/MS method for analysis, reported the level of DMT in whole rat brain (*n* = 2) taken from animals pretreated with a MAOI as being 10 and 15 ng/kg. This study's information is unfortunately quite limited in terms of sample number and did not address extraction recoveries, method validation or brain distribution of DMT.

As noted earlier, one study, using rat pups of different ages (1–40 days after birth) and conducted using a validated extraction/gas chromatographic-mass spectrometric analysis of whole-brain extracts (Beaton and Morris, 1984), examined the ontogeny of DMT in rat brain and found significant changes in the concentrations of DMT as a function of age. The highest levels were 17.5 ± 4.18 ng/g of brain (wet weight) at day 17. Values for other days ranged from undetected (limit of detection of 1.0 ng/g) to 1, 2 or as high as 11 ng/g. A *n* = 6 and a total of 4–6 brains were pooled for each day-post-birth analysis. Since pooled whole brain (2.0 g total weight) was used for the analysis, it is still not known how the DMT was distributed in the brain or if the DMT observed actually arose from a discrete brain area or areas alone. The data necessarily expressed the DMT concentration as if it was homogeneously distributed. Rats were also sacrificed at constant times during the study and no accounting was made for possible circadian or ultradian variations.

Given these facts, any speculation that attempts to dismiss the relevance of DMT *in vivo* because the concentrations in brain are too low (Nichols, 2017) necessarily ignores the fact that data concerning the actual levels of DMT in brain, particularly humans and levels that may be observed in different brain areas, simply does not exist.

### Future research to determine the concentration of DMT in brain tissues

While more research into the brain concentrations and distribution of DMT is obviously warranted, it is possible, as with many other substances, that it may only be found in specific brain areas or cell types. For example, the pineal gland of an adult rat weighs between 0.9 and 1.56 mg and the total brain weight is approximately 2.0 g. If all of the DMT found, on average, at day 17 (17.5 ng/g) in the Beaton and Morris (1984) study were to be located solely in the pineal, the tissue concentration would range between 18.9 and 10.9 ug/g or, converting ug to moles and gram to liter, the concentrations would be about 0.1 umoles/g or 0.1 mmoles/L to 0.06 umoles/g or 0.06 mmoles/L. While converting g to ml regarding tissue is by no means exact, the point to be made is that DMT in brain could have significant concentrations in discrete brain areas and exist in sufficient concentrations in such areas to readily affect various receptors and neuronal functions. Lower concentrations could occur in other brain areas as well with their concentrations being enhanced by mechanisms for DMT uptake and vesicular storage. What is obvious from these speculative calculations is the fact that more research into DMT brain distribution and concentrations is needed, recognizing its rapid metabolism and possible sequestration. It is quite clear that we have no good estimates at present concerning brain/neuronal distribution or concentration of endogenous DMT, particularly in humans, that will permit informed decisions or conclusions to be drawn regarding its function or the relevance of *in vitro* binding studies and relative Km's (Nichols, 2017) to endogenous levels. As with measurements in other matrices, well validated and sensitive methods for such quantitative analyses will be required.

## Receptor binding of DMT: 5-HT2A, TAARs, and sigma-1 receptors

There is a significant literature correlating the binding affinity of DMT and related hallucinogens for the 5HT2A receptor and its subset of receptors with other hallucinogens and their subsequent behavioral effects (Glennon et al., 1978; Nichols, 2004, 2016; Blough et al., 2014; Carbonaro and Gatch, 2016). However, DMT has been shown to interact with a variety of ionotropic and metabotropic receptors. While the subjective behavioral effects of exogenously administered DMT appear to be primarily acting via 5-HT2A receptors, the interaction of other receptors, such as other serotonergic and glutaminergic receptors, may also play a synergistic and confounding role. Indeed, the activation of frontocortical glutamate receptors, secondary to serotonin 5-HT2A receptor-mediated glutamate release, appears to be a controlling mechanism of serotonergic hallucinogens (dos Santos et al., 2016a,b). However, although this type of receptor research is quite mature, these findings have yet to define and accurately correlate what makes a compound hallucinogenic vs. compounds that have similar binding characteristics that are not hallucinogenic. Clearly, we are missing some pieces to the hallucinogen receptor/mode-of-action puzzle.

For example, Keiser et al. (2009) have shown that DMT binds to a variety of 5-HT receptors and that such binding does have physiological relevance. In their study, the role of 5-HT2A agonism in DMT-induced cellular and behavioral effects was examined in both cell-based and 5-HT2A knock-out mouse models. It was reported that “DMT binds to 5-HT1A, 5-HT1B, 5-HT1D, 5-HT2A, 5-HT2B, 5-HT2C, 5-HT5A, 5-HT6, and 5-HT7 receptors with affinities from 39 nM to 2.1 μM” (Keiser et al., 2009). Nonetheless, it was observed that DMT was not only a potent partial agonist at 5-HT2A but also that the DMT-induced head twitch response, a common measure of hallucinogenic activity, occurred only in wild-type mice but not in 5-HT2A knockout mice. However, it has been shown (Strassman, 1996) that the mixed 5-HT1A/1B antagonist pindolol markedly potentiates the subjective effects of DMT in humans. Furthermore, DMT-enhanced inositol trisphosphate production has been shown to persist even in the presence of the 5-HT2A antagonist ketanserin (Deliganis et al., 1991), suggesting other receptor sites for DMT's effects. Of interest is the finding of Urban et al. (2007) that receptors, such as the 5-HT family, can couple to multiple effectors, which allows receptor agonists to produce different pharmacological endpoints. Thus, certain compounds may selectively activate a specific subset of effectors producing a functional selectivity that complicates the interpretation of observed psychopharmacological or biochemical effects. In this regard, Carhart-Harris and Nutt (2017) have recently offered a novel bipartite model of serotonin neurotransmission involving co-modulation of the 5-HT1A and 5-HT2A receptors. This bipartite model purports to explain how different serotonergic drugs (including psychedelics) modulate the serotonergic system in different ways to achieve their observed pharmacology.

Clearly the 5-HT2A receptor is involved in the mode of action of DMT and other hallucinogens, but is it also clear that this is not the sole receptor on which we should rely for an overall explanation (Ray, 2010; Halberstadt and Geyer, 2011; Nichols, 2012, 2016).

Despite the failure of serotonin receptor binding theory to completely explain hallucinogenic activity, these observations support the 5-HT2A receptor as being a possible primary target for DMT's hallucinogenic effects (Keiser et al., 2009). While DMT has been shown to bind to the 5-HT2A receptor with relative high affinity (IC50 75 ± 1 nM; McKenna et al., 1990), many other compounds that lack DMT's visual effects have a higher affinity for the 5-HT2A receptor (McKenna et al., 1990).

In examining the possible complex interaction of multiple systems that may be necessary to explain the effects of compounds such as DMT, attention has also turned toward additional possible binding sites. Another set of functionally relevant binding sites for DMT is the family of trace amine-associated receptors (TAARs) (Burchett and Hicks, 2006; Wallach, 2009). DMT has been shown to be an agonist in binding to TAAR-1 with high affinity, causing activation of adenylyl cyclase and cAMP accumulation in TAAR1 transfected HEK293 cells. However, as is the case with the 5-HT2A receptor, other psychedelics and non-psychedelics also stimulate cAMP production following binding at TAAR1. There has yet to be sufficient research of TAAR to determine what role, if any, this class of receptors plays in the pharmacology or endogenous function of DMT. Thus, the research to date regarding the role of TAAR receptors suffers from the same lack of explanation for the mode of action of the hallucinogens as the 5-HT2A but may comprise a piece of what is obviously a complex set of interactions.

Another receptor family has also been implicated; the sigma-1 receptor. One of the possible roles of the sigma-1 receptor appears to be to act as an intracellular chaperone between the endoplasmic reticulum (ER) and mitochondria. In this role, it is involved in the transmission of ER stress to the nucleus (Carbonaro and Gatch, 2016). This process would be expected to result in the enhanced production of anti-stress and antioxidant proteins, with the activation of sigma-1 mitigating the possible damage done by hypoxia or oxidative stress (Szabo et al., 2014, 2016; Szabo and Frecska, 2016). Using *in vitro* cultured human cortical neurons (derived from induced pluripotent stem cells), monocyte-derived macrophages, and dendritic cells, Szabo et al. (2014) have shown that DMT greatly increases the survival of these cell types in severe hypoxia (0.5% O_2_), apparently via its interaction with sigma-1 receptors. A decreased expression and function of the alpha subunit of the hypoxia-inducible factor (HIF-1) was also observed, suggesting that DMT-mediated sigma-1 activation may alleviate hypoxia-induced cellular stress and increase survival via decreased expression and function of the stress factor HIF-1α in severe hypoxia. Such a mechanism has relevance to stroke, myocardial infarct or similar arterial occlusive disorders, cardiac arrest, and perinatal asphyxia, all conditions associated with hypoxic consequences (Carbonaro and Gatch, 2016). Szabo et al. (2016) and Szabo and Frecska (2016) have speculated that DMT may also contribute to neuroregenerative and neurorestorative processes by modulating the survival of microglia-like cells.

These sigma-1 associated effects may also be related to findings that DMT affects the rate of genetic transcription associated with synaptic plasticity (O'Donovan et al., 1999; González-Maeso et al., 2007), increased expression of brain-derived neurotrophic factor (BDNF) expression associated with synaptic plasticity (O'Donovan et al., 1999), cognitive processes such as memory and attention (DeSteno and Schmauss, 2008), and modulation of efficacy and plasticity of synapses (Soulé et al., 2006).

The sigma-1 receptor has been implicated in several neurobiological disorders and conditions and is found widely distributed though out the body, including in the CNS. However, both hallucinogens and non-hallucinogens bind to sigma-1 receptors, again complicating an attribution to this receptor as the primary site of DMT's action. Further, DMT binds to sigma-1 receptors at what should be considered as a high concentration (EC50 = 14 μM vs. about 75 nM for 5-HT2A) but does, nonetheless, have agonist activity. INMT has been shown to be co-localized with sigma-1 receptors in C-terminals of motor neurons (Mavlyutov et al., 2012) and such intracellular synthesis would allow for DMT accumulation and storage, producing the necessary μM concentrations for its action. It is also important to consider that the role of endogenous DMT is not necessarily to produce the same effects as observed from exogenous administration and such a “normal” role may be one of its biological assets.

It has also been observed that sigma-1 receptor agonists are potentially neuroprotective (Frecska et al., 2013). DMT has been shown to reduce neuronal inflammation via the sigma-1 receptor (Szabo et al., 2014) and can also induce neuronal plasticity, a long-term recuperative process that goes beyond neuroprotection (Tsai et al., 2009; Ruscher et al., 2011; Kourrich et al., 2012). Sigma-1 receptors can also influence cell survival and proliferation (Collina et al., 2013) and Frecska et al. (2013) have suggested that DMT is protective during cardiac arrest and perinatal development. With respect to the ontogeny of DMT, Lin et al. (1974) and Beaton and Morris (1984) have examined changes in INMT activity and DMT biosynthesis, respectively, with age in the rat. Taken together, changes in INMT levels consequently yielded increased DMT synthesis. It is possible that DMT-mediated sigma-1 receptor activity is also increased during this period to induce neuronal changes in newborns. Several selective sigma-1 receptor agonists have been shown to be protective against excitotoxic perinatal brain injury (Griesmaier et al., 2012) and ischemic neurodegeneration in neonatal striatum (Yang et al., 2010). In addition, it has been suggested that adequate expression of placental INMT may be necessary for pregnancy success (Nuno-Ayala et al., 2012).

### Future DMT receptor binding studies

Studies examining non-serotonergic receptors for DMT, such as TAAR and sigma-1, have begun to bear useful and insightful evidence for the possible “normal” roles of endogenous DMT and should be extended and expanded. Molecular biological studies of DMT's effects on these receptors and DMT's effects on their up-or-down regulation will also prove informative. Mapping of these receptors in brain tissues, with a determination of the nature and degree of colocalization of DMT's enzymes for synthesis in mind, will also add impetus to the growing recognition of DMT's possible “normal” functions in brain. This understanding may also lead to new therapeutic applications for regulating and altering endogenous DMT levels and function, providing new avenues for understanding hallucinogen pharmacology and their possible therapeutic use. The data suggest that the 5-HT2A receptor is only part of the story. The data further suggest there may well remain a “hallucinogen” receptor or receptor complex that has yet to be discovered. A more integrative mechanism to explain hallucinogenic activity, as suggested by Urban et al. (2007); Ray (2010); Halberstadt and Geyer (2011); and Carhart-Harris and Nutt (2017), is also intriguing and requires further inquiry.

Perhaps the true “hallucinogen” receptor has already been discovered and is simply mislabeled as being one of the many 5-HT receptors. Perhaps it is their interaction with many receptors and their complex functional connectivity that produces the observed effects (Ray, 2010; Halberstadt and Geyer, 2011). Indeed, the data suggest that DMT is both endogenous and possesses the properties of a neurotransmitter (see below). Studies have clearly shown that it binds with respectable affinity to the 5-HT2A receptor as well as other members of the serotonin family of receptors and elicits biochemical and physiological activity that can be correlated, to some degree, with such binding. These data support the idea that it is, therefore, an endogenous ligand for such receptors and intrinsically involved in serotonergic function. This being the case, there is already a significant body of work regarding DMT's binding and effects, especially relative to effects on serotonin, acting as a serotonergic modulator. Additional work in this area, while acknowledging DMT as an endogenous ligand, will prove essential. It is also unlikely that DMT acts alone in exerting it effects. Changes in relevant metabolomic and array profiles following DMT administration will further add to our understanding of its endogenous role.

## Administration of DMT

Szára (1956, 1961) originally reported that the effects of a medium dose (0.7 mg/kg) of DMT, given intramuscularly, were similar to those of mescaline and LSD, including visual illusions and hallucinations, distortion of body image, speech disturbances, mood changes and euphoria or anxiety (dependent on set and setting). Several other studies have replicated these findings using either IV or IM administrations (Turner and Merlis, 1959; Rosenberg et al., 1964; Gillin et al., 1976; Strassman et al., 1994a,b). Intramuscular effects of DMT at a reported dose of 0.2–1 mg/kg (Szára, 2007) generally had a rapid onset (2–5 min) and lasted 30–60 min. The IM effects are usually less intense than intravenous or inhalation-of-vapor routes of administration.

The subjective effects of DMT from ayahuasca administration (0.6–0.85 mg/kg DMT; Riba et al., 2003) usually appear within 60 min, peak at 90 min and can last for approximately 4 h (Cakic et al., 2010). The prolongation of effect is attributed to the MAOI effects of the constituent harmala alkaloids. Riba et al. (2015) have also reported the effects of oral and vaporized DMT alone. As expected, oral ingestion of pure DMT produced no psychotropic effects. Vaporized DMT was found to be quite psychoactive. This study also showed that smoked DMT caused a shift from the MAO-dependent route to the less active CYP-dependent route for DMT metabolism. Commonly used doses for vaporized or inhaled free-base DMT are 40–50 mg, although a dose may be as much as 100 mg (Shulgin and Shulgin, 1997). The onset of vaporized DMT is rapid, similar to that of i.v. administration, but lasts less than 30 min. It is of interest to note that intranasal free-base DMT is inactive (0.07–0.28 mg/kg; Turner and Merlis, 1959) as is DMT administered rectally (De Smet, 1983).

There is also additional significant literature concerning the administration of DMT via consumption of ayahuasca. While of great scientific interest, this subject is not reviewed here. This is mainly due to the complexity of composition of ayahuasca, especially the presence of significant MAOI effects.

Strassman et al. (1994a,b) have reported dose-response data for intravenously administered DMT fumarate's neuroendocrine, cardiovascular, autonomic, and subjective effects in a group of experienced hallucinogen users. DMT was administered at doses of 0.05, 0.1, 0.2, and 0.4 mg/kg to 11 experienced hallucinogen users. The results of these studies showed peak DMT blood levels and subjective effects were attained within 2 min after drug administration and were negligible at 30 min. DMT was also shown to dose-dependently elevate blood pressure, heart rate, pupil diameter, and rectal temperature, in addition to elevating blood concentrations of β-endorphin, corticotropin and cortisol. Prolactin and growth hormone levels rose equally at all doses of DMT. Levels of melatonin were unaffected. The lowest dose that produced statistically significant effects relative to placebo and that was also hallucinogenic was 0.2 mg/kg (Strassman, 1991, 1996).

The effects observed and the biochemical and physiological parameters measured in these studies add needed insight into the role and function of endogenous DMT. However, we must distinguish the effects of exogenously administered DMT from that which may be observed from its natural role as an endogenous substance. Exogenous administration of a bolus of DMT represents an “overdose” of a naturally occurring compound that may, when administered in this manner, exert a more complex pharmacology. However, this could also be true of any physiological change that produced a “normal” elevation in endogenous DMT, such as a response to stress or hypoxia, but with the entire process still remaining under a greater degree of biochemical control and response and the elevation possibly occurring in only certain brain areas or systems. For exogenously administered DMT we know plasma concentrations between 12 and 90 ng/ml (Callaway et al., 1999; Yritia et al., 2002; Riba et al., 2003) must be attained in order to produce hallucinogenic effects. The concentrations actually attained in whole brain or in specific areas required to produce hallucinogenic effects from such administrations are unknown.

### Future DMT administration studies

While these “overdoses” have given us valuable data regarding DMT's pharmacology and hints as to DMT's normal role and function, it will be necessary to lower the doses and expose the brain only to more “natural” levels or ranges to more fully ascertain why DMT is in the brain and what it is doing there. Part of that research will require the renewal of drug administration studies to assess the many prospects that have been raised by recent and current research. Gallimore and Strassman (2016) have offered an interesting proposal regarding the future conduct of DMT administration research; a target-controlled continuous, low-dose, IV infusion. This approach would be conducted to better discern the physiology and pharmacology of DMT and to produce a “prolonged and immersive psychedelic state.” The short duration of DMT's effects prevents the use of single dose administration as the research model for such studies. Target-controlled continuous IV infusion is a technology developed to maintain a stable brain concentration of anesthetic drugs during surgery. The rationale for this approach and the conduct of such research lies in the fact that DMT users have consistently reported “the complete replacement of normal subjective experience with a novel ‘alternate universe,’ often densely populated with a variety of strange objects and other highly complex visual content, including what appears to be sentient ‘beings.”' A further stated purpose of this approach, and one that would be quite informative, is to allow greater functional neuroimaging of the DMT experience, with subjects remaining under the influence of DMT for the extended periods necessary to collect the best data.

The administration of DMT by the IV route will require determination of an effective continuous dose, such that the desired level of experience is both attained and maintained. The lower the dose necessary the less likely volunteers will be to experience some of DMT's other peripheral and central “side-effects” and will establish a threshold above which further higher dose administrations may be examined. Concomitant administration of a MAOI would assist in attaining this goal but has the drawback of affecting levels of many other amine neurotransmitters as well, complicating the effects and subsequent data interpretation.

However, one alternative method of administration may be to use analogs of DMT that are structurally altered as so to inhibit the ability of the molecule to be metabolized by MAO-A, such as an alpha methyl or 2-N, N-dimethyl-propyl sidechain structure. However, such molecules may not bind in the same manner as DMT itself and may have other untoward effects. Another alternative that may assist in the ability to use lower doses and to prolong the effect of the DMT administered, however, may be the use of a deuterated analog.

In 1982, Beaton et al., reported on the behavioral effects of DMT and α,α,β,β-tetradeutero-DMT (**9**, Figure 3; D_4_DMT) administered interperitoneally to rats at a dose level of 2.5 and 5.0 mg/kg. The D_4_DMT was observed to produce, at equivalent doses to DMT itself, a significantly greater disruption of behavior, a longer duration of action and a shorter time to onset than non-deuterated DMT. This potentiation was apparently due to the kinetic isotope effect which, in theory, makes it harder for the MAO enzyme to extract a deuterium (vs. a hydrogen) from the alpha position (Figure 3), thus inhibiting degradation by MAO. In a companion study, Barker et al. (1982) also showed that, at the same dose, D_4_DMT attained a significantly higher brain concentration than DMT itself and that the elevation in brain level lasted for a longer period of time. Similar data have recently been presented for a tetra deutero-5-MeO-DMT (Halberstadt et al., 2012) and the authors reached a similar conclusion; these results demonstrate that deuterated tryptamines may be useful in behavioral and pharmacological studies to mimic the effects of tryptamine/MAOI combinations, but without the MAOI. While the synthesis of deuterated analogs may be more expensive initially, newer methods for such synthesis (Brandt et al., 2008) may overcome these concerns. Furthermore, the pharmacological properties of D_4_DMT may render it orally active. Such a possibility has yet to be explored. It is also possible that oral administration and kinetic isotope effect inhibition of metabolism may prolong the effects of a deuterated analog sufficiently to also be of use in imaging studies.

It would be of interest to determine if the proposal of Gallimore and Strassman (2016), using a continuous infusion of DMT, would also be of use in in an animal model for the treatment of severe brain injury and trauma or in conditions resulting from a hypoxic insult, such as arterial occlusive disorders, cardiac arrest, and perinatal asphyxia, promoting the possible neuroprotective and neuroregenerative effects of DMT that have been recently described. Such studies will also allow validation or refutation of the recent data in this area.

## Imaging research

While there have been several studies reporting neuroimaging data from volunteers consuming ayahuasca (Bouso et al., 2015; dos Santos et al., 2016a,b), there is minimal neuroimaging data for the administration of DMT alone (Daumann et al., 2008, 2010). Using functional magnetic resonance imagining (fMRI) techniques, administration of DMT “caused a decreased blood oxygenation level-dependent response during performance of an alertness task, particularly in the extrastriate regions during visual alerting and in temporal regions during auditory alerting.” (Daumann et al., 2010). It was concluded that the effects for the visual modality were more pronounced. Imaging data for other hallucinogens, such as psilocybin and LSD, have been generated (dos Santos et al., 2016b). dos Santos et al. (2016b) have concluded that “the acute effects of hallucinogen administration, as interpreted from imaging studies, included excitation of frontolateral/frontomedial cortex, medial temporal lobe, and occipital cortex, and inhibition of the default mode network.” For long-term use, the administration of hallucinogens was associated with “thinning of the posterior cingulate cortex, thickening of the anterior cingulate cortex, and decreased neocortical 5-HT2A receptor binding.” It was also suggested that hallucinogens “increase introspection and a positive mood by modulating brain activity in the fronto-temporo-parieto-occipital cortex” (dos Santos et al., 2016b).

### Future imaging research

The data to be derived in such imaging studies are highly dependent on the instrumentation and methods used and the interpretation of the data can often be somewhat subjective. However, any such data may provide the necessary roadmaps to understand brain distribution of administered and endogenous DMT and the activation-deactivation profiles created naturally or artificially in various states of consciousness. Indeed, recent imaging data (Carhart-Harris et al., 2012, 2016; Tagliazucchi et al., 2014) and pharmacological studies of 5-HT2A receptor activation (Kraehenmann, 2017; Kraehenmann et al., 2017) suggest that hallucinogens create a brain-image patterning that resembles dream states. Such studies of DMT have yet to be reported and should be undertaken. The involvement of DMT in various dream states has been hypothesized (Callaway, 1988). One possible mechanism is the possibility that endogenous DMT is the signaling molecule responsible for the up-and-down regulation of specific brain areas that occurs during different dream states. Understanding the DMT-related functional connectivity or connectome, either from administration and/or from endogenous production stimulation, will expand our research frontiers in this field. Administration studies, such as proposed by Gallimore and Strassman (2016), could provide imaging data that will permit interpretation of the neural pathways relevant to DMT's effects, particularly in eliciting hallucinations, but also as part of its “normal” function.

## DMT as a neurotransmitter, neurohormone, or neuroregulatory substance

In 1976, Christian et al., published the accumulated evidence that DMT was a naturally occurring transmitter in mammalian brain, having met the criteria for such a designation at the time; “1) the synthetic enzymes and substrates are present in the CNS for the production of DMT, 2) a binding site is present to react with the compound and 3) the compound is found in human CSF and isolated synaptic vesicles from rat brain tissue” (Christian et al., 1976). Additional criteria have been added over the years, such as demonstration of electrophysiological activity. Indeed, DMT had also been shown to change the transepithelial and intracellular potentials of the blow-fly salivary gland and to increase the production of cyclic AMP (Berridge, 1972; Berridge and Prince, 1974) early on. Another added criterion is that a pathway for DMT's metabolism and removal must be demonstrated. Pathways of DMT metabolism in the brain are well understood (Barker et al., 1980) and newer data offers other mechanisms, such as uptake into synaptic vesicles and neurons, for controlling its synaptic levels (Cozzi et al., 2009; Blough et al., 2014). Like any neurotransmitter, uptake and storage can allow a reservoir of DMT to remain stored in vesicles, ready for release, and provide a mechanism for protecting and concentrating the compound (Frecska et al., 2013).

Christian et al. (1977) subsequently described a specific high-affinity (Kd = 30 nM) binding site for DMT on purified rat synaptosomal membranes that was also sensitive to LSD but not to serotonin. DMT was also shown to lead to the production of cAMP in synaptosomal membrane preparations as well as in rat brainstem slices and rat cerebrum *in vivo* (Christian et al., 1977). Unfortunately, no additional research on these findings has been reported. Other studies have also demonstrated that administered DMT becomes localized in the synaptosomal fraction of rat brain following administration and is detected in the vesicular fraction of such preparations (Barker et al., 1984). Further, the Mg^2+^ and ATP dependent uptake of DMT (Corbett et al., 1975) into rat brain vesicles has also been demonstrated as has apparent high and low affinity uptake sites for active transport of DMT in rat brain cortical slices.

The supporting data for DMT as a neurotransmitter have continued to accumulate. DMT has also been shown to be taken up into neuronal cells via serotonin uptake transporters (SERT) on neuronal plasma membrane (Berge et al., 1983; Whipple et al., 1983; Nagai et al., 2007; Cozzi et al., 2009) and Cozzi et al. (2009) have shown sequestration of DMT into synaptic vesicles from the cytoplasm by the neuronal vesicle monoamine transporter 2 (VMAT2). Blough et al. (2014) have also shown that DMT releases 5-HT via SERT with an EC50 in the low nM range. This indicates that DMT is a substrate for the SERT transporter and provides a further mechanism for the neuronal accumulation of DMT. Newer data concerning INMT in specific brain areas (Cozzi et al., 2011) and its presence in perfusates of the pineal gland of living rats (Barker et al., 2013) add additional evidence for DMT's potential role as a neurotransmitter. At a minimum, the anatomy, pharmacology and physiology of DMT have been sufficiently characterized and demonstrated to afford DMT the classification as a putative neurotransmitter.

The concentration of DMT into vesicles and its release at the synaptic cleft would permit elevated concentrations of DMT, perhaps sufficient to elicit its known pharmacological actions as well as other effects. It would also be protected from MAO degradation. Peripheral production of DMT would not be required. It may also be the case that brain DMT biosynthesis is inducible in response to specific physiological effects, causing an increase in concentration in specific cell types and areas. This being the case, the idea that a pharmacologically relevant blood level of DMT must be attained before such effects are observed (Nichols, 2017) from endogenous production of DMT would not be relevant.

### Future studies characterizing DMT as a neurotransmitter

Setting aside speculation in favor of what has been scientifically proven, the effects of administered psychedelics must be recognized as acting via existing, naturally occurring, neuropharmacological pathways and mechanisms. Perhaps we should first consider research into the possible role of endogenous DMT in explaining the elusive mode of action of the varied class of compounds possessing hallucinogenic properties. There is no doubt that DMT acts on the serotonergic system as well as other known neurotransmitter systems. Nonetheless, if DMT is a neurotransmitter, neurohormone and/or neuroregulatory substance then we should consider all of the more well understood properties of agonists and antagonists acting on such a system. While many hallucinogens have been shown to act on many different neurotransmitters and receptors, we may now add the need to examine their effects on the synthesis, binding, release, reuptake, storage, degradation, etc. of an “endogenous hallucinogen,” DMT. This is especially true in relation to serotonin regulation. As with our more recent understanding of the mode of action of opiates, finding new endogenous ligands and receptors can actually lead to a more complete understanding of the effect of what often appear to be divergent substances. Hypothetically, the mode of action of hallucinogens may be via their effects on an endogenous hallucinogen neuronal system (Barker et al., 1981). Establishing DMT as a neurotransmitter makes such research not only somewhat obvious and relevant but necessary. If such a system is found to be responsible for these phenomena it may lead to more discoveries explaining other normal or pathological conditions such as, for example, delirium, certain symptoms of psychoses, spontaneous hallucinations and sleep disorders, autism and other perceptual anomalies. Perhaps it may yet be shown to be involved in schizophrenia, just not necessarily by previously expressed mechanisms. Certainly, it could give us insight into the proposals of its involvement in our more human attributes of creativity, imagination and dream states (Callaway, 1988) and of our less common experiences of visions, NDEs and extraordinary states of consciousness occurring without exogenous administration of a hallucinogenic substance. Thus, we need to better understand the molecular biology, physiology and anatomy surrounding endogenous DMT and its potential regulatory role.

Taken together, the evidence for DMT as a neurotransmitter is compelling. Recent research and more classical data have established that it is synthesized, stored, and released in the brain and mechanisms for its uptake, metabolism and removal have all been established. While more work remains to establish DMT as a neurotransmitter, such as more electrophysiological and iontophoretic data, it appears to be following the same path to recognition as other neurotransmitters have followed before final acceptance (Carlsson, 2001).

## DMT as a therapeutic

There has been a renewed interest in using hallucinogenic drugs as therapeutics in clinical research to address depression (Berman et al., 2000; Aan het Rot et al., 2010; Buchborn et al., 2014), obsessive-compulsive disorder (Moreno and Delgado, 1997), the psychological impacts of terminal illness (Grof et al., 1973; Grob et al., 2011), prisoner recidivism (Hendricks et al., 2014), and substance abuse disorders, including alcohol (Bogenschutz et al., 2015) and tobacco (Mangini, 1998; Krebs and Johansen, 2012). Most of these studies have examined the use of LSD, psilocybin or ayahuasca instead of DMT alone.

In the history of use of DMT-containing “remedies,” ayahuasca has perhaps the longest record (Dos Santos et al., 2011; Alonso et al., 2015; Pic-Taylor et al., 2015). Long-term use of ayahuasca has been shown to produce measurable changes in the brain itself, such as differences in midline brain structures as determined from MRI studies (dos Santos et al., 2016a,b). While such effects may not appear to be of therapeutic value, long-term ayahuasca users (>10 years) have shown reduced ratings of hopelessness (Santos et al., 2007). Long-term ayahuasca use has also produced marked improvement in depressive symptoms with no concomitant mania or hypomania for up to 21 days after a single dose (Osório et al., 2015). These data suggest evidence for a potential antidepressant effect for DMT. However, ayahuasca is a complex mixture containing MAOIs (harmala alkaloids) which, as a class of drugs, have also been used alone to treat depression. Thus, it is impossible to say from such studies that DMT itself or the elevation of other brain neurotransmitters in combination is responsible for the perceived positive clinical effects or even if the hallucinations produced by DMT consumed under these conditions are themselves somehow cathartic.

While other classic hallucinogens (LSD, psilocybin, etc.) are beginning to show promise in the treatment of addictions (drugs, alcohol, etc.) as well as post-traumatic stress (PTSD) and other mental disorders (Bogenschutz and Pommy, 2012) there has yet to be generated conclusive evidence regarding the efficacy of DMT in any of them. DMT has been shown to exert anti-anxiety/anti-psychotic properties at the trace amino acid receptor (TAAR) and others have suggested that the possible positive symptoms observed in schizophrenia may be mediated by the effects of endogenous DMT (Cakic et al., 2010; Grammenos and Barker, 2015). These findings do not necessarily support the conclusion that DMT is useful for treatment of anxiety or mental illness, however. The possible use of DMT as an adjunct to psychiatric therapy has been proposed by numerous investigators, a proposal that contravenes the tenets of the transmethylation hypothesis.

Frecska et al. (2013) have suggested that DMT may be involved in significant adaptive mechanisms that can also serve as a promising tool in the development of future medical therapies and there have been proposals that DMT might be useful to treat substance abuse, inflammation, or even cancer. However, at this point, the necessary data to support such proposals have not been presented and it would be premature to propose that DMT will become commonly used for clinical purposes. If it is a neurotransmitter, then understanding its role and function in normal or disease states could provide pharmacological targets to alter these functions, however.

### Future study of DMT as a therapeutic

At present, the data arguing for the use of DMT as a therapeutic, particularly via administration, is thin. The claimed therapeutic effects for DMT in combination with harmala MAOIs as in ayahuasca or pharmahuasca (Ott, 1999) is of interest but presents a complex data set that prevents an understanding of the contribution of each component. To further study DMT without the effects of an MAOI, research should pursue whether or not D_4_DMT is orally active, as previously noted, which would enhance the opportunities to examine its potential as a therapeutic. The use of hallucinogens in psychotherapy is gaining renewed interest and certainly DMT should be among the drugs in the psychiatric pharmacopeia. Any proposal to pursue this avenue will require more than the current combined body of scientific evidence. Both Federal and State laws will have to change in order to make the manufacture and use of such compounds easier and to make conducting the necessary research feasible.

However, if DMT is a neurotransmitter and is responsible for modulation of serotonergic or other neurotransmitter systems, it may well be that many existing pharmaceuticals already exert their pharmacology via DMT-related-effect mechanisms. This may be the case for the other hallucinogens, as noted, but may also be true for part of the mode of action of certain serotonergic drugs, such as antidepressants. Further characterization of DMT cellular distribution, receptors and general biochemistry may lead to new targets for more effective pharmaceutical substances and interventions.

## Conclusions

It has been 86 years since DMT's first synthesis by Manske and 61 years since Szara discovered its hallucinogenic properties. It has been 41 years since Christian et al. characterized DMT as a neurotransmitter. Further research has better defined the latter's characteristics such that a compelling case can be made, at a minimum, to consider DMT as a putative neurotransmitter.

Over time, the observations of the hallucinogenic phenomena experienced following the administration of DMT have led to speculation that endogenous DMT is possibly involved in psychosis, normal attributes and experiences such as creativity, imagination and dream states, maintenance of waking reality, altered states of consciousness including religious and/or spiritual phenomena, and NDEs. Even more far reaching and “other worldly” hypotheses have also been offered, suggesting that DMT, as well as other hallucinogens, may provide actual proof of and/or philosophical insights into many of our unanswered questions regarding extraordinary states of consciousness. Regardless of the level and cause of such speculation and hypotheses, it is only scientific research that can inform or refute such thinking. There is no doubt that hallucinogen research has been a forbidden fruit long ripening on the tree of knowledge.

Recent research has demonstrated that DMT is present in and is released from the pineal gland of live, freely-moving rodents. Although older data suggested that DMT might not be synthesized to any great extent in brain, studies have now shown that the necessary enzymatic components for the biosynthesis of DMT are present in discreet brain cell types and areas as well as other tissues not previously examined. New receptors for DMT have been identified and a potential role for DMT as a neuroprotectant and/or neuroregenerative agent has been suggested. Hallucinogens have been shown to produce brain patterning resembling dream states, apparently mediated through 5-HT2A receptor activation. DMT's effect in this regard has yet to be examined, but raises speculation as to one of the possible roles of endogenous DMT.

As discussed and delineated above, more research is needed on DMT's natural role and function and interaction with other neurotransmitter systems. This will require the recommended future research into DMT biosynthesis, metabolism and binding, new methods for peripheral and central detection and data from administration, imaging and therapeutic trial studies. The data derived from the areas of research addressed above will no doubt suggest several possible new avenues for additional future research on DMT. In order to advance, however, regulatory blockades to hallucinogen research must be removed. Progress in hallucinogen research in these areas has been slowed due to over-regulation. For at least the last 50 years, research on DMT and other hallucinogens has been impeded in the United States by passage of the Congressional Amendment of 1965 and the Controlled Substances Act of 1970 by the United States Congress that classified DMT and other major hallucinogens as Schedule-I substances. Given the endogenous nature of DMT, it deserves a special status for future research.

It is evident that we have too long ignored the field of hallucinogen research, in all of its potential aspects. This is especially true if continuing research demonstrates a clear role for one of its more prominent members, DMT, as an endogenous regulator of brain function. It is my opinion that these and many other possible approaches and hypotheses regarding DMT and other psychedelics are research endeavors that have great potential and are worthy of attention and support. Turning the newest technologies to this work, in genetics, analytical chemistry, molecular biology, imaging and others, we will no doubt acquire both new knowledge and ask new questions. If the politics of any one nation forbid it, perhaps others will take up the challenge to further the knowledge of our own potential and the further development and understanding of what we prize as our most unique human characteristic; the untapped possibilities of the mind.

## Author contributions

The work presented is the sole effort of SB who agrees to be accountable for all aspects of the work in ensuring that questions related to the accuracy or integrity of any part of the work are appropriately investigated and resolved.

### Conflict of interest statement

The author declares that the research was conducted in the absence of any commercial or financial relationships that could be construed as a potential conflict of interest.
